# Added sugars and risk of osteoarthritis in adults: A case-control study based on National Health and Nutrition Examination Survey 2007–2018

**DOI:** 10.1371/journal.pone.0313754

**Published:** 2024-11-14

**Authors:** Xiaolong Liao, Xiali Chen, Yumei Zhou, Limin Xing, Yihua Shi, Guoxin Huang

**Affiliations:** 1 Department of Orthopedics, Xiangyang No.1 People’s Hospital, Hubei University of Medicine, Xiangyang, Hubei, China; 2 Department of Nursing, Xiangyang No.1 People’s Hospital, Hubei University of Medicine, Xiangyang, Hubei, China; 3 Department of Evidence-Based Medicine Center, Xiangyang No.1 People’s Hospital, Hubei University of Medicine, Xiangyang, Hubei, China; Sri Ramachandra Institute of Higher Education and Research (Deemed to be University), INDIA

## Abstract

**Objective:**

Added sugars have been associated with a variety of adverse health consequences, but their relationship with osteoarthritis is unclear. This study aimed to demonstrate the association between added sugars and osteoarthritis.

**Methods:**

We used the National Health and Nutrition Examination Survey (NHANES) database from 2007 to 2018 to explore the association between added sugars and osteoarthritis.

**Results:**

In our study, 2,746 adults were included. The average age of the chosen participants was 43.77 years, with 52.33% males and 47.67% females. There were 2,152 in the osteoarthritis group and 594 in the non-osteoarthritis group, weighted to represent 11,854,966 participants. In the fully adjusted multivariable model 3, added sugars were found as a risk factor for osteoarthritis (OR = 1.01; 95% CI 1.00 to 1.01), with populations in the fourth quartile having a greater prevalence of osteoarthritis (OR = 1.40; 95% CI 1.09 to 1.81). When added sugars were treated as a continuous variable in subgroup analysis, the results indicated that never consumed alcohol (OR = 1.02; 95% CI 1.01 to 1.04) and no history of diabetes (OR = 1.02; 95% CI 1.01 to 1.04)were more Likely tend to osteoarthritis. When added sugars were treated as a categorical variable in subgroup analysis, the results indicated that compared to the first group, in the fourth quartile population, females (OR = 1.44; 95% CI 1.02 to 2.02), low BMI (OR = 1.88; 95% CI 1.06 to 3.33), never smoking (OR = 1.55; 95% CI 1.05 to 2.30), never consumed alcohol (OR = 3.31; 95% CI 1.42 to 7.74), no history of hypertension (OR = 1.51; 95% CI 1.00 to 2.27), and no history of diabetes (OR = 1.44; 95% CI 1.11 to 1.87) were more likely tend to osteoarthritis.

**Conclusion:**

Added sugars are a risk factor for osteoarthritis, especially in females, low BMI, never smoking, never consumed alcohol, no history of hypertension, and no history of diabetes.

## Introduction

Osteoarthritis (OA) is a bone and joint degenerative disease that causes cartilage injury, hyperostogeny, subchondral bone sclerosis, and synovial inflammation [1]. OA currently affects over 300 million people worldwide, with approximately 10% of men and 18% of women over the age of 60 suffering pain and physical impairment [2,3]. While there are few therapy choices for OA, patients with the disease can delay its progression by practising appropriate self-management.

Food plays a vital role in promoting health and avoiding disease and there is evidence that diet is closely connected to the development of OA [4]. As a vital and fundamental diet component, sugar is an important energy supplier for our bodies. Added sugars are defined as sweeteners added during food processing and preparation, excluding natural sugars found in vegetables, fruits, and milk [5]. Types of added sugars include brown sugar, cane juice, dextrose, fructose, fruit nectar, high fructose corn syrup, honey, lactose, maltose syrup, raw sugar, and sucrose [6]. The primary sources are sugary beverages (regular soft drinks, sweetened tea and coffee, energy drinks, and juice), candies, and desserts (cakes, cookies) [7]. Although the Dietary Guidelines for Americans (DGA) have recommended that Americans aged ≥2 years consume less than 10% of their total daily calories from added sugars and that children aged <2 years should avoid added sugars [8], excessive added sugars intake remains a global public health problem. Research has indicated that those who consume substantial quantities of added sugars are more likely to have weight gain and have an increased susceptibility to conditions such as cancer, diabetes mellitus(DM), dyslipidemia, hypertension, Parkinson’s disease, and cardiovascular disease(CVD) [7,9,10].

Added sugars have been related to a range of adverse health consequences, but whether there is a link between added sugars and OA is still unknown. As a result, we examined the connection between added sugars and OA using data from a large sample of the National Health and Nutrition Examination Survey (NHANES).

## Materials and methods

### Population sources

NHANES is a cross-sectional, population-based survey that collects data on the health and nutrition of the US household population. The investigation, which began in the 1960s, mostly comprises interview and physical examination data. The survey has been conducted on a two-year cycle since 1999, with approximately 5000 individuals chosen for data collection each year. We examined NHANES data from six consecutive cycles spanning 2007 to 2018, including information on OA and added sugars.

This project was approved by the National Center for Health Statistics (NCHS) Research Ethics Review Board and all participants. This cross-sectional study followed the STROBE standards for reporting observational studies in epidemiology [11].

The primary outcome was whether the participants had OA or not. In this study, OA was defined by participant self-report. Participants who answered "Yes" to the question "Have you/sample person (SP) ever had osteoarthritis?" in the NHANES questionnaire section. Participants who replied "yes" were considered to have OA.

### Added sugars assessment

The primary exposure factor is added sugars. In NHANES, added sugars include brown sugar, cane syrup, corn syrup, corn syrup solids, dextrose, fructose, fruit syrup, honey, maple syrup, molasses, pancake syrup, raw sugar, sorghum syrup, and white sugar. We used 24-hour dietary recall to estimate added sugars intake. Participants from NHANES were all allowed to participate in two 24-hour dietary recalled interviews. The first dietary recall interview occurred face-to-face at the Mobile Exam Centre (MEC), and the second interview was completed by telephone within 3 to 10 days. Dietary data (including total energy and added sugars) was extracted from the US Department of Agriculture Pyramid Equivalent Database/Food Pattern Equivalent Database (MPED/FPED) files based on total nutrient intake on the first and the second day. The teaspoon equivalent (tsp) values of FPED added sugars were converted to grams (4.2 g/tsp) and kcal (3.87 kcal/g).

### Covariates assessment

The following covariates were included in the participants: Age, gender (male and female), race (white, Mexican, black, other race), marital status (married, divorced, widowed, separated, never married, living with partner), education level (under high school, high school or equivalent, above high school), poverty, Variables of laboratory data such as TG (mmol/L), TC (mmol/L), HDL (mmol/L), LDL (mmol/L), Medical examination and personal life history such as physical activity, BMI, alcohol intake (never, former, mild, moderate, heavy), smoking (never, former, now), Comorbidities such as cancer (yes or no), diabetes (yes or no), hypertension (yes or no).

### Statistical analysis

We used complex sampling weights recommended by the Centers for Disease Control and Prevention (CDC). Following the method recommended on the NHANES website, we combined the sample weights for six consecutive cycles. First, we delineated into quartile groups (Q1-Q4) according to participants’ added sugars intake: Q1 [0.000, 7.444], Q2 [7.444, 13.942], Q3 [13.942, 24.016], and Q4 [24.016, 263.793]. In the baseline characteristics table, continuous variables with normal distributions were described by the mean (SD), continuous variables with skewed distributions were described by the median (IQR) and analyzed using a t-test or rank-sum test for statistical inference; the categorical variable was described using the rate, and compared with the chi-square test. Second, we utilized multivariate logistic regression to investigate the connection between added sugars and OA, with the lowest quartile as the reference for all covariates. Model 1 was an unadjusted weighted logistic regression; Model 2 was adjusted according to age, gender, and race/ethnicity; and Model 3 had adjustments for all covariates. Finally, we performed subgroup analyses using gender (male, female), age (≦60 years, >60 years), BMI (low, normal, high), smoking (never, former, now), alcohol intake (never, former, mild, moderate, heavy), diabetes (yes or no), and hypertension (yes or no) as stratification factors.

## Results

### General baseline characteristics of participants

**Fig 1:**
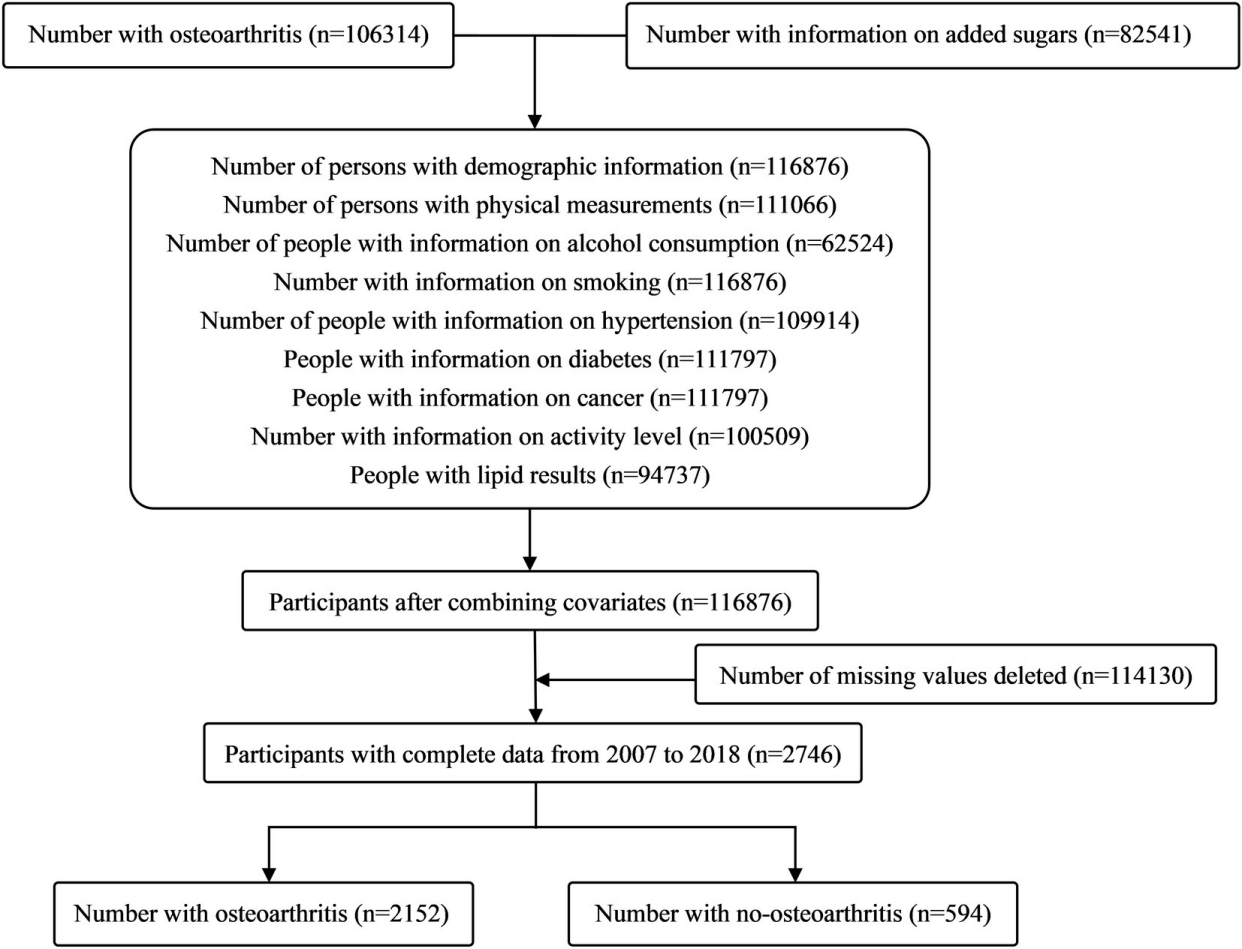
Flowchart of the study populationl.

Fig 1 displays the study population’s inclusion and exclusion criteria(see Fig 1). Table 1 shows baseline characteristics for overall participants and four quartiles(see Table 1). 106,314 participants were evaluated for OA in the NHANES from 2007–2018, and 82,541 participants had data related to added sugars intake. After adjusting for variables, 116,876 participants were included. Participants with missing values were excluded, resulting in the inclusion of 2,746 participants. After weighting, 11,854,966 participants can be represented. There were 52.33% males and 47.67% females, the weighted mean age is 43.77 years old, the mean BMI is 28.15, the median household income is 3.19, and 88.92% of the participants had no OA, and 11.08% had OA.

**Table 1:** Weighted selected characteristics of study population in female and male, NHANES (Weighted N = 118549666).

Character	Total	Q1	Q2	Q3	Q4	P value
BMI, mean(SD)	28.15(0.09)	28.31(0.17)	27.73(0.16)	28.32(0.17)	28.25(0.14)	0.01
Age, mean(SD)	43.77(0.27)	46.62(0.48)	45.90(0.37)	43.60(0.42)	39.35(0.39)	< 0.0001
Poverty, median(IQR)	3.19(0.03)	3.37(0.05)	3.33(0.04)	3.22(0.05)	2.86(0.05)	< 0.0001
Physical Activity,median(IQR)	1280.00(433.65,3780.00)	1320.00(480.00,3360.00)	1260.00(480.00,3360.00)	1320.00(444.85,3840.00)	1225.00(378.00,5040.00)	0.6
TG(mmol/L),median(IQR)	1.12(0.78,1.65)	1.11(0.75,1.61)	1.08(0.77,1.63)	1.10(0.76,1.61)	1.17(0.85,1.74)	< 0.0001
TC(mmol/L),median(IQR)	4.91(4.27,5.61)	4.97(4.29,5.69)	4.99(4.32,5.74)	4.89(4.27,5.53)	4.86(4.22,5.51)	< 0.001
HDL(mmol/L),median(IQR)	1.34(1.11,1.63)	1.45(1.19,1.76)	1.40(1.16,1.68)	1.32(1.11,1.61)	1.22(1.03,1.45)	< 0.0001
LDL(mmol/L),median(IQR)	2.92(2.35,3.54)	2.90(2.30,3.52)	2.92(2.35,3.57)	2.90(2.35,3.52)	2.97(2.38,3.57)	0.24
Alcohol intake, %						< 0.0001
Never	9.24	10.30	8.86	10.08	7.85	
Former	11.41	10.46	9.24	11.26	14.48	
Mild	38.72	38.63	41.63	38.71	36.04	
Moderate	18.32	21.09	18.84	18.38	15.29	
Heavy	22.31	19.52	21.44	21.57	26.34	
Cancer, %						0.003
No	92.65	92.01	91.06	92.94	94.47	
Yes	7.35	7.99	8.94	7.06	5.53	
Gender, %						< 0.0001
Male	52.33	42.48	46.00	51.68	67.76	
Female	47.67	57.52	54.00	48.32	32.24	
Race/Ethnicity, %						< 0.0001
White	71.91	71.71	72.63	71.24	72.04	
Mexican	7.25	6.53	6.88	7.90	7.65	
Black	9.58	6.66	8.39	11.00	11.97	
Other	11.26	15.10	12.10	9.86	8.33	
Marital status, %						< 0.0001
Married	56.97	59.62	58.28	58.14	52.27	
Living with partner	8.34	6.82	8.06	8.10	10.19	
Separated	2.03	1.76	1.64	1.93	2.74	
Divorced	9.24	8.81	9.71	8.30	10.08	
Widowed	3.39	4.42	4.31	3.30	1.70	
Never married	20.02	18.57	18.01	20.22	23.02	
Education, %						< 0.0001
Under high school	12.01	10.05	10.43	11.77	15.48	
High school or equivalent	22.74	18.48	19.08	23.20	29.58	
Above high school	65.25	71.47	70.49	65.04	54.94	
Diabetes, %						< 0.0001
No	93.71	89.30	92.75	94.99	97.35	
Yes	6.29	10.70	7.25	5.01	2.65	
Hypertension, %						< 0.0001
No	69.21	64.02	68.66	71.19	72.51	
Yes	30.79	35.98	31.34	28.81	27.49	
Smoking, %						< 0.0001
Never	55.67	57.88	56.35	57.90	50.94	
Former	24.11	27.10	26.75	24.33	18.72	
Now	20.22	15.02	16.90	17.77	30.33	
OA, %						< 0.001
No	88.92	86.69	87.59	89.95	91.20	
Yes	11.08	13.31	12.41	10.05	8.80	

SD: Standard Deviation, IQR: Interquartile Range, BMI: Body mass index, TG: Triglycerides, TC: Total cholesterol, HDL: High-density lipoprotein, LDL: Low-density lipoprotein.

### Logistic regression analysis results

In the logistic regression analysis, we assessed the impact of added sugars on the risk of OA using both continuous and categorical variable approaches. When added sugars were treated as a continuous variable in multiple logistic regression analysis, the results showed that in model 1, OR = 0.98 (95% CI 0.98 to 0.99; P < 0.0001), suggesting a slight decrease in the risk of OA with increased added sugars. In model 2, OR = 1.01 (95% CI 1.00 to 1.01; P = 0.04), indicating an increase in risk. In model 3, OR = 1.01 (95% CI 1.00 to 1.01; P = 0.03), reflecting a continued upward trend in risk. When added sugars were treated as a categorical variable in multivariate logistic regression analysis, the results showed that the Q4 group had a greater risk of OA than the Q1 group in the fully adjusted model 3 (P = 0.01; OR = 1.40, 95% CI 1.09 to 1.81), indicating a 40% increase in risk for the Q4 group (see Table 2).

**Table 2:** Weighted ORs (95% CIs) for the associations between added sugars and osteoarthritis across three models.

Exposure	Model 1^a^	P Value	Model 2^b^	P Value	Model 3^c^	P Value
Add sugars (%kcal)	0.98(0.98,0.99)	**<0.0001**	1.01(1.00,1.01)	**0.04**	1.01(1.00,1.01)	**0.03**
Quartile of %kcal added sugars						
Q1	Ref	Ref	Ref	Ref	Ref	Ref
Q2	0.92(0.76,1.12)	0.42	0.97(0.78,1.20)	0.75	1.02(0.82,1.26)	0.88
Q3	0.73(0.57,0.93)	**0.01**	0.97(0.74,1.26)	0.79	1.01(0.78,1.31)	0.92
Q4	0.63(0.51,0.78)	**<0.0001**	1.35(1.06,1.73)	**0.02**	1.40(1.09,1.81)	**0.01**

Ref: Reference.

^a^Non-adjusted model: Adjusted for none.

^b^Minimally adjusted model: Adjusted for age, gender, and race/ethnicity.

^c^Fully adjusted model: Adjusted for age, gender, race, marital status, education level, poverty, Laboratory variables (TG, TC, HDL, LDL in mmol/L), physical activity, BMI, alcohol, smoking, cancer, diabetes, hypertension.

### Subgroup analysis results

When added sugars were treated as a continuous variable in subgroup analysis, the results indicated that never consumed alcohol (OR = 1.02; 95% CI 1.01 to 1.04) and no history of diabetes (OR = 1.01; 95% CI 1.00 to 1.01) were more likely tend to OA(see Table 3).

**Table 3:** Stratified logistic regression analysis.

Character	Add sugars (%kcal)	Quartile of %kcal added sugars			
		Q1	Q2	Q3	Q4
Gender					
Male	1.01(1.00,1.02)	Ref	0.97(0.68,1.39)	1.00(0.71,1.41)	1.30(0.88,1.93)
Female	1.01(1.00,1.01)	Ref	1.03(0.78,1.36)	1.07(0.78,1.48)	**1.44(1.02,2.02)** *
Age					
= <60	1.00(0.99,1.01)	Ref	1.02(0.73,1.43)	0.96(0.68,1.36)	1.09(0.79,1.51)
>60	1.01(0.99,1.02)	Ref	0.99(0.74,1.31)	0.98(0.67,1.43)	1.37(0.84,2.24)
BMI					
Normal	1.01(0.99,1.02)	Ref	0.78(0.53,1.15)	1.08(0.70,1.65)	1.14(0.75,1.75)
Low	1.01(1.00,1.02)	Ref	1.19(0.77,1.84)	1.02(0.64,1.61)	**1.88(1.06,3.33)** *
High	1.01(0.99,1.02)	Ref	1.13(0.77,1.66)	0.96(0.63,1.46)	1.38(0.90,2.12)
Smoke					
Never	1.01(1.00,1.02)	Ref	0.93(0.68,1.28)	0.98(0.67,1.45)	**1.55(1.05,2.30)** *
Former	1.00(0.98,1.01)	Ref	0.90(0.61,1.31)	0.93(0.61,1.43)	0.95(0.58,1.56)
Now	1.01(1.00,1.02)	Ref	1.86(0.97,3.58)	1.23(0.57,2.68)	1.72(0.90,3.30)
Alcohol					
Never	**1.02(1.01,1.04)** **	Ref	**2.04(1.05,3.96)** *	1.90(0.77,4.70)	**3.31(1.42,7.74)** **
Former	1.00(0.99,1.01)	Ref	1.20(0.67,2.12)	1.58(0.86,2.90)	1.56(0.80,3.06)
Mild	1.01(1.00,1.02)	Ref	0.89(0.62,1.30)	0.86(0.58,1.28)	1.26(0.85,1.86)
Moderate	1.00(0.98,1.02)	Ref	1.10(0.61,2.00)	1.10(0.65,1.85)	1.09(0.50,2.36)
Heavy	1.01(0.99,1.02)	Ref	0.80(0.35,1.85)	0.66(0.30,1.48)	1.37(0.71,2.64)
Hypertension					
No	1.00(1.00,1.01)	Ref	1.36(0.95,1.94)	1.04(0.70,1.53)	**1.51(1.00,2.27)** *
Yes	1.01(1.00,1.02)	Ref	0.79(0.60,1.04)	1.03(0.73,1.44)	1.36(0.95,1.94)
Diabetes					
No	**1.01(1.00,1.01)** *	Ref	1.04(0.82,1.32)	1.05(0.79,1.39)	**1.44(1.11,1.87)** **
Yes	1.01(0.98,1.04)	Ref	0.84(0.49,1.44)	0.71(0.32,1.55)	1.20(0.44,3.24)

Ref: Reference, BMI: Body mass index.

*P<0.05

**P<0.01.

When added sugars were treated as a categorical variable in subgroup analysis, the results indicated that compared to group Q1, in group Q4, female (OR = 1.44; 95% CI 1.02 to 2.02), low BMI (OR = 1.88; 95% CI 1.06 to 3.33), never smoking (OR = 1.55; 95% CI 1.05 to 2.30), never consumed alcohol (OR = 3.31; 95% CI 1.42 to 7.74), no history of hypertension (OR = 1.51; 95% CI 1.00 to 2.27), and no history of diabetes (OR = 1.44; 95% CI 1.11 to 1.87) were more likely tend to OA(see Table 3).

## Discussion

Healthy eating patterns exclude excess added sugars, and there are different opinions on the daily maximum for added sugars. The World Health Organization (WHO) suggests limiting free sugars to less than 10% of total daily calorie consumption, with extra health benefits associated with restricting to less than 5% of total daily energy intake, or around 25g per day [12]. The Institute of Medicine (IOM) recommends that added sugars make up less than 25% of total calories [8]. According to the NHANES, sugar-sweetened beverages were the most popular item consumed by participants, with the highest added sugars intake among all age groups, owing to participants’ lack of awareness of the possibly harmful consequences of added sugars [10]. Current evidence suggests that excessive intake of added sugars is associated with an increased risk of non-alcoholic fatty liver disease, cardiovascular disease, diabetes, and obesity [9,13]. It is unknown whether there is an association between added sugars and OA. Therefore, we analysed the relationship between added sugars intake and OA to give new evidence for a link.

This cross-sectional study is the first to investigate the relationship between added sugars and OA risk by analyzing six consecutive cycles of the NHANES dataset. The results suggest that added sugars are a risk factor for OA. The gut microbiota plays a crucial role in regulating bone metabolism, which is significant for the development of OA [14]. High sugar intake reduces gut microbial diversity, leading to dysbiosis, which is associated with chronic low-grade inflammation, a known factor in the development of osteoarthritis [15,16]. For example, High consumption of sugars decreases microbial diversity and reduces luminal short-chain fatty acids (SCFAs). SCFAs can affect the recruitment of colonic regulatory T cells and the antibacterial activity of macrophages, thereby influencing the intestinal mucosal immune system [17,18]. The damaged intestinal barrier cannot prevent the invasion of pathogenic microorganisms, allowing bacterial endotoxins to enter the bloodstream and trigger systemic inflammation. This chronic inflammation could further degrade joint cartilage, contributing to OA progression. In addition, excessive sugar consumption can result in metabolic disorders and elevate inflammatory mediators, along with specific pro-inflammatory cytokines in diverse organs [19]. For example, Excessive intake of added sugars promotes the translocation of microbial constituents from the intestine to the portal vein, thereby activating the NF-κB and JAK2/STAT3 pathways through TLR4, which leads to the production of inflammatory markers such as IL-6 and TNF-α [20,21]. These inflammatory factors may contribute to the progression of osteoarthritis by facilitating cartilage degradation via the activity of matrix metalloproteinases (MMPs) [22]. Moreover, added sugars contribute to the development of metabolic syndrome, a condition closely linked to OA [23]. The increased oxidative stress from excessive sugar intake further damages joint tissues by promoting reactive oxygen species (ROS) production, accelerating cartilage degradation [24,25]. Although the exact biological mechanisms linking added sugars and OA remain to be fully elucidated, current evidence suggests that excess sugar-induced changes in gut microbiota, inflammation, and metabolic and oxidative pathways may play crucial roles. Future studies should dissect these pathways more clearly to provide targeted interventions for reducing OA risk through dietary modifications.

In addition, our study found a new insight: female, low BMI, never smoking, never consumed alcohol, no history of hypertension, and no history of diabetes were more likely to develop OA when added sugars intake was >24.016 kcal. We suggest that this gender difference can be explained by the role of basal metabolic rate (BMR) in energy expenditure and its relationship with excess added sugar consumption. Basic metabolic rate typically accounts for 60–70% of total daily energy expenditure, and it plays a critical role in maintaining energy balance within the body [26]. Research indicates that men tend to have a higher BMR compared to women. For example, Francisco et al [27]. discovered that men have a BMR of 1548 ± 305 kcal/d, whereas women have a significantly lower BMR at 1098 ± 301 kcal/d. This suggests that men are more efficient at metabolizing excess sugar, potentially burning off excess energy more easily than women. However, gender differences in the susceptibility to OA may also be influenced by other factors beyond BMR alone. Studies have shown that estrogen levels significantly drop after menopause in women, leading to critical changes in the subchondral bone. These changes, such as damage to bone biomechanical structures, often coincide with or even precede cartilage degradation, which may increase the risk of osteoarthritis in women or accelerate its progression [28]. In addition, gender-related cellular molecular and genetic differences (e.g., higher levels of genetic markers of inflammation in women), and anatomical differences (e.g., bone size, shape, and rotation) further explain the higher prevalence of osteoarthritis in women [29]. Surprisingly, our study discovered that patients with a low BMI, never smoking, never consumed alcohol, no history of hypertension, and no history of diabetes were more likely to develop OA. Studies have shown that micronutrient deficiencies caused by nutritional deficiencies may affect OA because these micronutrients play an important supporting role in various metabolic processes and immune functions of joint tissues [30]. Patients with low BMI are typically deficient in nutrients required by the body, whereas high added sugars consumption provides relatively little nutritional benefit. After consuming excessive amounts of added sugars, participants may not have the extra room to consume other nutrients needed in a healthy diet, and the body’s nutritional status further deteriorates, which may be related to the fact that low BMI are more susceptible to OA. Furthermore, the study discovered no significant link between smoking, alcohol consumption, hypertension, diabetes, and OA when excessive quantities of added sugars were taken in. While these lifestyle factors and comorbidities are known to contribute to various adverse health outcomes, their relationship with OA, particularly in the presence of high added sugars intake, did not show a significant link in our analysis.Although there is not enough data to elucidate why, Our guess is is hypothesized that individuals who already have poor lifestyle habits or disease states may have adapted to the negative effects of these unfavourable factors. In such cases, when excessive amounts of added sugars are consumed, the body may not exhibit a pronounced "stress response", added sugars may have more of a bystander effect at this time. The term "bystander effect" here refers to the possibility that added sugars do not directly intensify the effects of these other risk factors but rather act in a more secondary or indirect capacity. This implies that the consumption of added sugars does not trigger an additional harmful response beyond what is already caused by other lifestyle-related factors, essentially making their impact seem secondary or "bystander-like" during the interaction with these pre-existing conditions.

Our study has several benefits. First, while our study was cross-sectional rather than prospective, we were able to provide insight into the association between added sugars and the prevalence of OA by analysing a large nationally representative sample. Second, we accounted for a wide range of potentially confounding variables in our study to ensure the reliability of our findings. These strengths provide a reliable foundation for our study, enable us to understand the effects of added sugars on OA more accurately, and provide valuable references and insights for future related studies.

There are several potential limitations of the current study. First, due to the cross-sectional nature of the study, we were unable to establish a causal relationship between added sugars intake and the prevalence of OA. While we found an association between the two, it is unclear whether high added sugars intake leads to OA or if individuals with OA tend to consume more added sugars. The inability to determine the temporal sequence between these variables significantly limits our ability to draw causal inferences. Second, although OA and dietary intake data in the NHANES database were collected through self-report questionnaires with rigorous quality control, recall bias remains a concern. Participants may inaccurately report their food consumption, including added sugars intake, leading to misclassification and measurement error, potentially affecting the observed association between added sugars and OA. Underreporting due to social desirability or difficulty recalling foods could weaken the relationship, while overreporting, though less common, could exaggerate it. NHANES mitigated this with two 24-hour dietary recall interviews and the use of standardized measures from USDA’s FPED, but self-reported data remain susceptible to bias. Future studies with more objective measures, like biomarkers, could reduce bias and improve causal inference. In addition, even though we adjusted for some possible confounders, we still could not eliminate the potential confounding effects of some unknown variables. In addition, the lack of data in the NHANES database on the prevalent sites of OA, such as the knee, hip, and spine, made it impossible to assess the relationship between different sites and added sugars. Finally, because different food sources of added sugars may differ in composition, energy density, and absorption, the present study did not provide a detailed categorization of food or beverage sources of added sugars or a breakdown of the types of added sugars, such as monosaccharides, disaccharides, or other sugars, and thus could not clarify the relationship between different sources and different classifications of added sugars and OA.Together, these limitations suggest that while our findings are suggestive, they should be interpreted with caution, and further research is needed to confirm the direction and strength of the relationship between added sugars and OA.

## Conclusions

In summary, this study provides cross-sectional evidence of the relationship between added sugars and OA. It suggests that we need to emphasize the negative health effects of added sugars and take measures to control the intake of added sugars in order to effectively manage and prevent the development of the disease. In the future, we should further conduct more high-quality prospective studies to elucidate the causal relationship between them. Such studies will provide a more reliable scientific basis for the development of more effective preventive and intervention measures to ensure people’s health and well-being.

## Supporting information

S1 Data (XLSX)
